# Juvenile Hormone Is Required in Adult Males for *Drosophila* Courtship

**DOI:** 10.1371/journal.pone.0151912

**Published:** 2016-03-22

**Authors:** Thilini P. Wijesekera, Sumit Saurabh, Brigitte Dauwalder

**Affiliations:** Department of Biology and Biochemistry, University of Houston, Houston, TX, United States of America; Alexander Fleming Biomedical Sciences Research Center, GREECE

## Abstract

Juvenile Hormone (JH) has a prominent role in the regulation of insect development. Much less is known about its roles in adults, although functions in reproductive maturation have been described. In adult females, JH has been shown to regulate egg maturation and mating. To examine a role for JH in male reproductive behavior we created males with reduced levels of Juvenile Hormone Acid O-Methyl Transferase (JHAMT) and tested them for courtship. JHAMT regulates the last step of JH biosynthesis in the Corpora Allata (CA), the organ of JH synthesis. Males with reduced levels of JHAMT showed a reduction in courtship that could be rescued by application of Methoprene, a JH analog, shortly before the courtship assays were performed. In agreement with this, reducing JHAMT conditionally in mature flies led to courtship defects that were rescuable by Methoprene. The same result was also observed when the CA were conditionally ablated by the expression of a cellular toxin. Our findings demonstrate that JH plays an important physiological role in the regulation of male mating behavior.

## Introduction

Effective male courtship behavior is essential for successful reproduction in most animals and the study of this behavior allows important insight into the regulation of complex behaviors. In *Drosophila*, male mating behavior is well described and can easily be observed and quantified (for reviews see [1–3]. Significant insight into the brain neuronal circuits that control the behavior has been gained in the last decade [4–7]. In *Drosophila*, the sex-specific circuits of brain neurons are not determined by sex hormones as it is in mammals, but cell-autonomously by a series of alternative splicing events that result in the generation of the two male-specific transcription factors Fruitless (FRU) and Doublesex (DSX). FRU and DSX are required to establish the neuronal competence of the circuits that are required for male courtship behavior [6, 8–10]. However, these circuits are not sufficient for full normal courtship behavior. The adult fat body, a secretory tissue outside the brain, produces sex-specific factors that significantly contribute to normal mating behavior [11]. One of these factors, called Takeout, is secreted into the circulating hemolymph and has been shown to act in courtship as a secreted protein [12]. Takeout is similar to Juvenile Hormone Binding Proteins (JHBPs) from other insects based on sequence similarity and their characteristics of soluble carrier proteins. JHBPs bind Juvenile Hormone (JH), one of the major developmental insect hormones [13–15]. In adult females, JH is required for oogenesis [16–19] and JH has recently been shown to regulate the onset of female mating behavior and pheromone maturation [20]. In addition, JH has been shown to affect aging [21]. Mutations that affect JH titers or signaling have been shown to affect male mating behavior: It has been shown that a mutation in the gene *apterous* (*apt*) leads to a decrease in JH titers, a delay in the maturation of the fat body and male courtship defects [22–24] but it is unknown why *apt* mutants have lowered JH levels and whether this is the cause for the reduced courtship. *Methporene tolerant (Met)* is a transcription factor that has been shown to bind JH [25]. *Met* mutant males have mating defects that could partly be rescued by a *Met* transgene [26, 27]. Manipulation of JH levels during developmental stages by application of the JH analogue Methoprene can also result in courtship defects [28]. Together, these findings suggest a role for the hormone in courtship. However, since JH has a prominent role during development, it is unclear to what extent the described adult effects on courtship might be caused by altered levels of JH during development or brain maturation, since these mutations affect larval and adult stages alike. In this paper we sought to specifically examine the adult role of JH in the regulation of male courtship behavior. To this end, we conditionally expressed an RNAi transgene targeting JHAMT, one of the last enzymes in the JH biosynthetic pathway [29], in adult, mature males and observed a significant reduction in male courtship. The courtship defects were rescued by the application of Methoprene, a well-described JH analog, shortly before testing. Our data support an adult physiological role for JH in *Drosophila* male courtship behavior.

## Materials and Methods

### Fly strains

All fly strains were reared on standard corn meal/sugar-based medium at room temperature under non-controlled light conditions, except for *Gal80*^*ts*^ flies that were grown at 18°C and induced as adults at 30°C as indicated. The JHAMT RNAi line *UAS-* CG17330 *RNAi* ^103958^ was obtained from the VDRC stock collection, Vienna. *hsp70-Gal4*, *UAS-lacZ* and *UAS-DTI* strains were a gift from Gregg Roman, University of Houston.

### Creation of *JHAMT-GAL4* line

5043 bp of the promoter region upstream of the translation start of *JHAMT* (CG 17330) was PCR-amplified and inserted upstream of *GAL4* into the Not1 and BamH1 restriction sites of the pPTGAL vector (*Drosophila* Genomics Resource Center). The primers were: 5’-*ATA AGA ATG CGG CCG* CTG CGG TTT AGG GGT GCT ATG ACT-3’ and 5’- GC*G GAT CCC* TCG ACA ACT GAT CGA CGA TTG GGA C- 3’ (restriction enzyme sites in italics). The plasmid was injected into w^1118^ flies by Rainbow Transgenic Flies, Inc. (http://www.rainbowgene.com/), and transgenic lines established.

### Immunohistochemistry

Cryosections of whole flies were fixed in 4% Paraformaldehyde and immunohistochemistry performed as described in [30]. Double staining using anti-ßGal and anti-JHAMT antibodies was performed on the sections. The anti-JHAMT antibody raised in rabbit was a kind gift of Ryusuke Niwa (University of Tsukuba, Japan) [29] and was used at a 1:100 dilution. The mouse anti-ßGal antibody (Sigma) was used at 1:200. The secondary antibodies were Alexa-546 goat anti-rabbit antibody (1:200) and Alexa-633 goat anti- mouse at (1:200) (Invitrogen). Fluorescent preparations were viewed using an Olympus FV1000 confocal microscope, images were processed using Adobe Photoshop.

### Behavioral assays

The courtship assay and activity assay were performed as previously described [12] and as described in the text. For all assays, control and experimental flies were grown, collected and aged in parallel. In each behavioral session, complete sets of flies were tested. The number of tested flies (n = 20) was equal for all genotypes in an experiment.

### Gal80^ts^ experiments

For *Gal80*^*ts*^ experiments, control and experimental flies were raised at 18°C. Virgin males were collected at eclosion and kept for 5–8 days at 18°C. Matured flies were then placed at 30°C for the times indicated in the text. All flies were let to rest for 1–2 hours at RT prior to the behavioral assay. Non-induced controls from 18°C were subjected to the same resting period of 1–2 hours at room temperature before testing.

### Heat induction of hsp70-Gal4

*hsp70-GAL4* was used to drive the expression of the *UAS-JHAMT-RNAi* construct conditionally in adults. The flies were raised at room temperature and newly emerged flies kept in individual vials for 4 days. The *hsp70-GAL4* driver was induced by placing the flies in pre-heated vials at 37°C for 1 hour in an incubator. The flies were then transferred to fresh vials and placed at room temperature for 4 hours to rest. They were then subjected to a courtship assay. Activity assays were performed for flies of the same genotype.

### Methoprene treatment of flies

0.5% Methoprene was prepared by dissolving Methoprene (ChemService) in 100% Acetone. The flies were anaesthetized on ice and treated with the Methoprene solution (1μ of solution was applied on the ventral surface of the abdomen using a micropipette). The control flies were treated with Acetone only. The Methoprene treated and untreated (Acetone treated) flies were placed at room temperature for 4 hours and subjected to the courtship assay.

### Statistical analysis

Statistical analysis was performed by one-way or two-way ANOVA and Bonferroni multiple comparison *post hoc* test. Statistical analyses were performed with Statview (Adept Scientifics, Bethesda, MD).

## Results

### Creation of JHAMT-Gal4 lines

**Fig 1:**
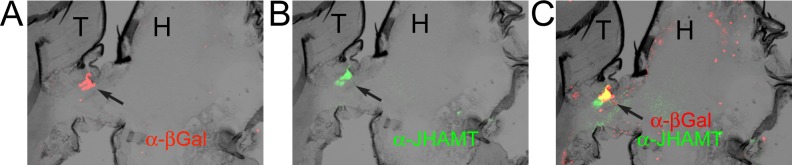
JHAMT-Gal4 driver directs expression in the corpora allata (CA). Frozen sections of adult *JHAMT-Gal4/UAS-lacZ* males were incubated with an anti-ßGal antibody (red) and with an anti-JHAMT antibody (green). Overlapping expression in the CA (marked by arrow) was observed. H:Head; T:Thorax

In order to reduce JH levels, we chose to target Juvenile Hormone acid O-methyltransferase (JHAMT), the enzyme that transfers a methyl group from S-adenosyl-L-methionine (SAM) to the carboxyl group of JH acids in one of the final steps of JH synthesis [29, 31]. A *Drosophila melanogaster* homologue *Dm*JHAMT (CG17330) has been characterized by Niwa et al. [29]. These authors have shown specific expression of the protein in the Corpora Allata (CA), the main place of JH synthesis. In order to drive expression of a *UAS-JHAMT RNAi* transgene specifically in the CA we generated a novel *JHAMT-Gal4* line by using 5 kb of upstream sequences of the JHAMT gene. To assess the expression of *JHAMT-Gal4* lines we crossed them to a *UAS-lacZ* containing strain. We observed prominent expression of ßGal in the CA, as confirmed by performing double-staining with anti-ßGal and anti-JHAMT antibodies [29] that showed significant overlap (Fig 1). Based on these staining we conclude that our new *JHAMT-Gal4* driver is well suited to target the CA.

### Reduction of JHAMT in the Corpora Allata (CA) leads to a courtship defect that can be rescued by adult application of the Juvenile Hormone analog Methoprene

**Fig 2:**
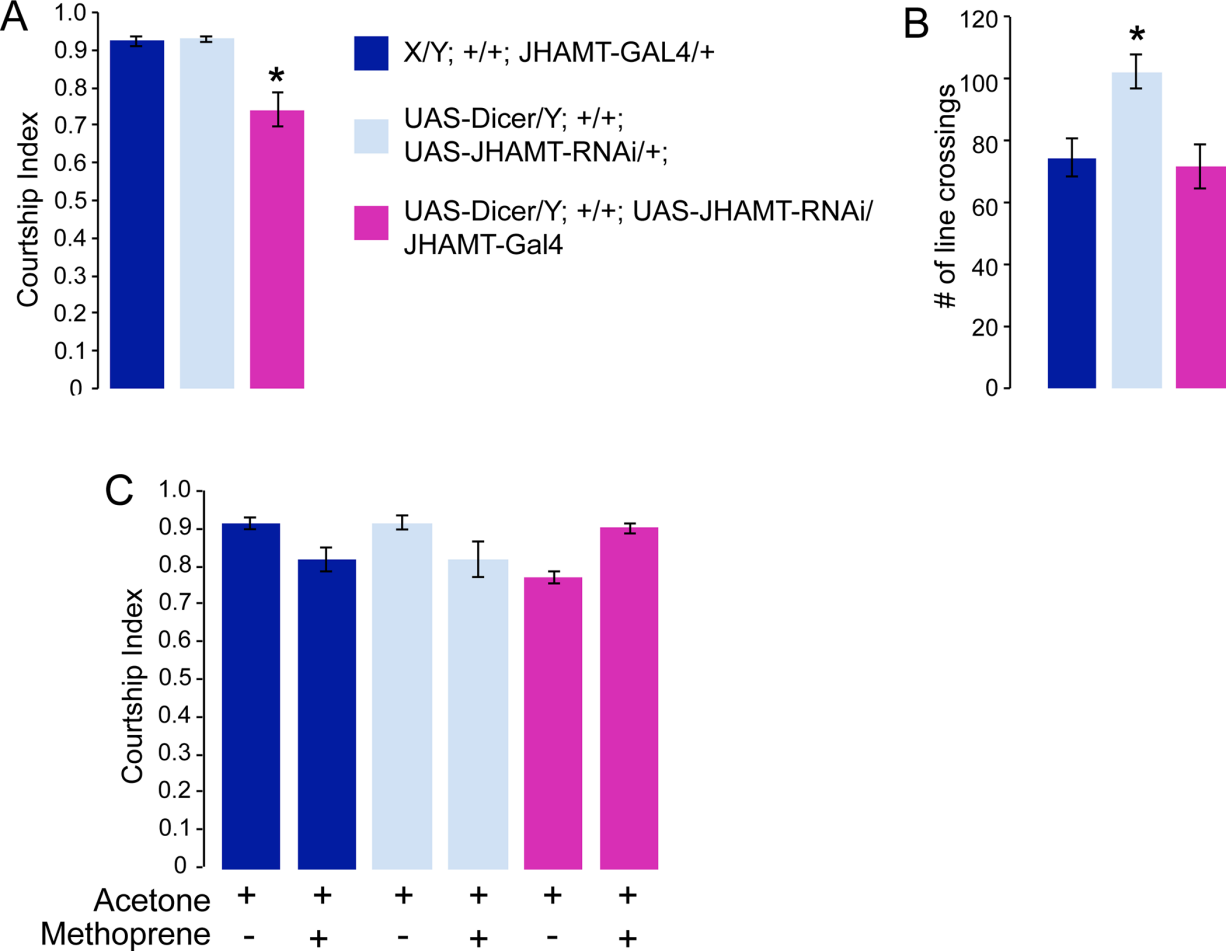
Expression of JHAMT-RNAi lowers the courtship index and can be rescued by application of the JH analog Methoprene. Graphs show the courtship index CI (fraction of time males spend courting during the observation period) ± SEM of the indicated genotypes (A, C), or the performance of males in a control activity assay (# of line crossings ± SEM) (B). Data were analyzed by ANOVA followed by Bonferroni multiple comparisons. A) Expression of *UAS-JHAMT RNAi* using our CA-specific *JHAMT-Gal4* driver significantly reduces male courtship in comparison to the control males. N = 20. (p < 0.001). Genotypes used were *UAS-dicer/Y; UAS-JHAMT-RNAi*/+; *JHAMT-GAL4/+;* and control genotypes *UAS-dicer/Y; UAS-JHAMT-RNAi/+; +/+* and *X/Y; +/+; JHAMT-GAL4/+*. B) Activity levels in the mutants and in control flies (genotypes as in A). Mutants are not different from the normally courting X/Y; +/+; *JHAMT-Gal4* control. (N = 10). C) Mature males of the genotypes described in (A) were treated with Methoprene in acetone or acetone alone, and tested four hours later. The experimental genotype shows complete rescue of the courtship index following Methoprene treatment compared to acetone treatment alone. (N = 20).

In order to reduce JHAMT in the CA, the main place of JH synthesis, we crossed *JHAMT-Gal4* flies to *JHAMT-RNAi* flies *(VDRC line* 103958). This line proved to be the strongest available RNAi line in our hands. We introduced an additional *UAS-dicer* transgene into experimental flies in order to enhance the effect of the RNAi transgene. Even in this combination the *JHAMT-RNAi* effect is weak, as suggested by the fact that *UAS-JHAMT-RNAi/+*; *JHAMT-Gal4/+* flies survive to adulthood. We did find, however, that RNAi expression driven by a strong *tubulin-Gal4* driver was lethal. For the courtship assays, we collected freshly eclosed males and aged them individually for 4 days before testing them in a courtship assay. In this assay, the male is paired with a single non-receptive virgin wildtype female in a mating chamber. His courtship behavior is then scored as the fraction of time he performs any of a series of well-defined courtship steps [1–3] within a 10-minute observation period. Experimental males carrying both the *JHAMT-Gal4* driver and the *UAS-JHAMT-RNAi* and *UAS-dicer* transgenes (*UAS-dicer/Y; UAS-JHAMT-RNAi/+; JHAMT-Gal4/+*) were tested alongside control flies that only carried either the JHAMT-Gal4 *(JHAMT-Gal4/+*) or the UAS-JHAMT-RNAi and UAS-dicer transgenes (*UAS-dicer/Y; UAS-JHAMT-RNAi/+*). The results are shown in Fig 2. *JHAMT-Gal4/UAS-JHAMT-RNAi* experimental males showed a significant decrease in courtship index (Fig 2A) in comparison to the genotype controls. This suggests that normal JH levels are required for normal male courtship behavior. To ascertain that the decrease in courtship is not just due to general sickness and sluggishness of the mutant males, we performed a short-term activity assay [32] that measures general locomotive activity. In this assay, males are grown, collected and aged as for the courtship assay. They are placed into the courtship chamber that has a line drawn across the bottom. The number of times the male crosses this line during the observation time is recoded (Fig 2B). One of the controls (*UAS-dicer/Y; UAS-JHAMT-RNAi/+*) has an activity index that is statistically higher than the other control (*X/Y; JHAMT-GAL4/+*) (p = 0.0045) and the experimental genotype (*UAS-dicer/Y; UAS-JHAMT-RNAi/JHAMT-GAL4*) (p = 0.0021). However, the activity index of the experimental genotype is not different from one of the controls that is normal in courtship, making it unlikely that the courtship defect is caused by general sickness of the experimental flies. Expression of JHAMT-RNAi in the CA is predicted to lower JH levels. To confirm that the observed courtship defect is due to a reduction in JH, we performed rescue experiments by applying Methoprene, an established JH analog [27]. Males were aged for 4 days, anesthetized on ice and 1 μl of 0.5% Methoprene dissolved in acetone was applied to their abdomen. As a control, acetone only was applied in parallel to another set of flies. Four hours after application, the males were tested in the courtship assay. As shown in Fig 2C, application of acetone alone did not affect the behavior of control males, and did not change the reduced courtship index of *UAS-dicer/Y; UAS-JHAMT-RNAi/+; JHAMT-GAL4/+* males. In contrast, application of Methoprene to *UAS-dicer/Y; UAS-JHAMT-RNAi/+; JHAMT-GAL4/+* males resulted in the complete rescue of the courtship defect, confirming lowered levels of JH as the cause of the reduced courtship observed in these animals. The rescue was obtained by application of Methoprene to mature flies shortly before subjecting them to the courtship assay. These results indicate an adult physiological requirement for normal JH levels in male courtship behavior. Application of Methoprene did not affect the courtship of control males, although we have observed a trend towards lower scores following Methoprene application. The knockdown of JHAMT mRNA by RNAi appears to be weak, since it did not affect the viability of these flies. A strong reduction of JH might have been expected to result in precocious metamorphosis [33, 34]. JH levels in larvae and adults are comparable [35, 36], suggesting that courtship behavior is more sensitive to relatively small JH disturbance than earlier developmental processes are.

### JH is required in adult males for courtship behavior

**Fig 3:**
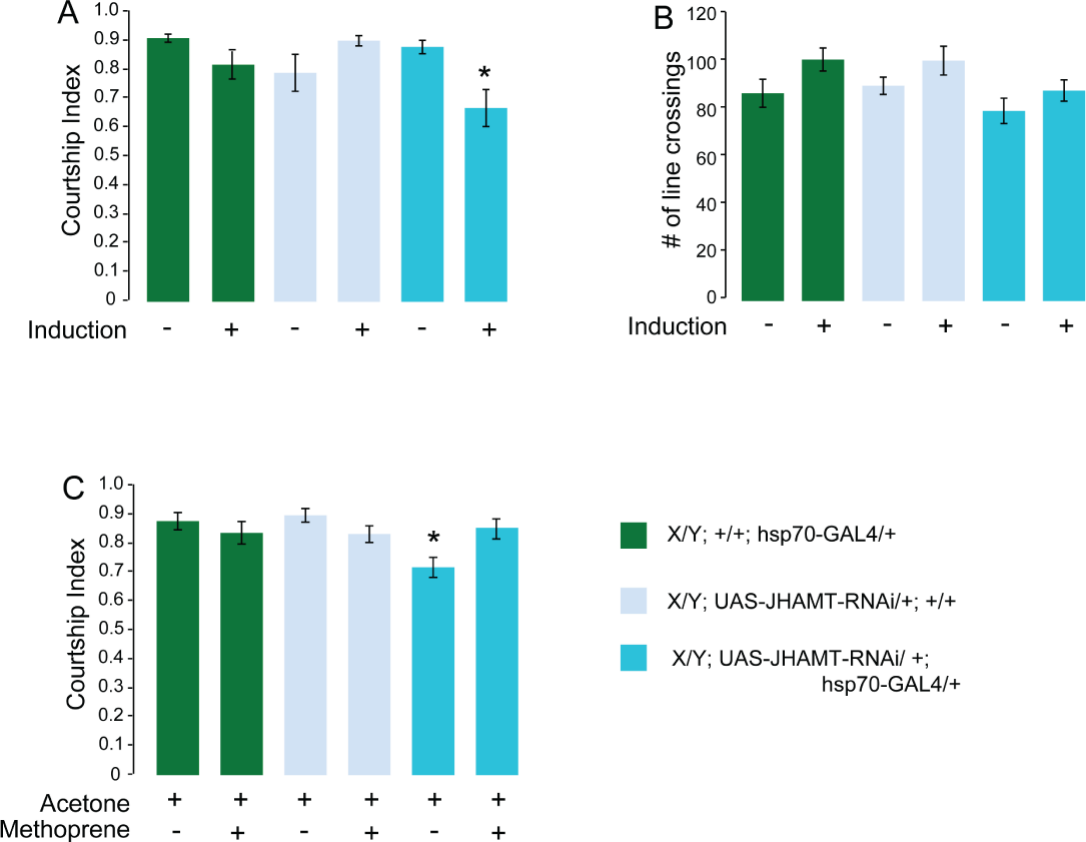
Conditional reduction of JHAMT in mature males causes courtship defects that can be rescued by application of the JH analog Methoprene. Graphs show the courtship index CI (fraction of time males spend courting during the observation period) ± SEM of the indicated genotypes (A, C), or the performance of males in a control activity assay (# of line crossings ± SEM (B). Data were analyzed by ANOVA followed by Bonferroni multiple comparisons. (A) Courtship index of induced and un-induced experimental (*X/Y; UAS-JHAMT-RNAi/+; hsp70-GAL4/+*) and control (*X/Y; +/+; hsp70-GAL4/+;* and *X/Y; UAS-JHAMT-RNAi/+;+/+*) genotypes. The experimental genotype shows a significant reduction in courtship index (p = 0.0007). (N = 20). Induced flies were heat-shocked at 37°C for 1 hour and let recover for four hours. (B) Activity assay of the induced and un-induced genotypes; genotypes as in (A) (n = 10). (C) Genotypes as in (A); one hour after induction, flies were treated with Methoprene in acetone, or with acetone alone, and courtship was examined 4 hours later. The experimental genotype shows complete rescue (p< 0.003) (N = 20). (Un-induced (-), Induced (+).

The fact that the courtship reduction of *UAS-dicer/Y; UAS-JHAMT-RNAi/+; JHAMT-GAL4/+* males can be rescued by the adult application of Methoprene strongly argues that the observed courtship defect is not due to developmental effects of JH reduction in these flies. To confirm that normal levels of JH are an important physiological adult component of courtship regulation, we next performed experiments in which we conditionally reduced JH levels only in mature adult flies. We used the heat-inducible ubiquitous *hsp70-Gal4* driver to conditionally express JHAMT-RNAi in mature males. 4-day-old males were subjected to a one-hour 37°C heat shock, let recover for four hours at room temperature and then tested for courtship. Non-induced flies were kept at room temperature and tested in parallel for comparison. As shown in Fig 3A, heat shock did not affect the courtship of the control *+/hsp70-Gal4* and *+/UAS-JHAMT-RNAi* flies. In contrast, it significantly reduced the courtship of *hsp70-Gal4/UAS-JHAMT-RNAi* males. Un-induced *hsp70-Gal4/UAS-JHAMT-RNAi* experimental males courted normally. To ascertain that the courtship defect we observed was due to JH reduction, we heat-shocked experimental and control flies as described above and applied either Methoprene or the vehicle alone one hour after heat shock. The flies were then tested for courtship four hours later. Again, application of Methoprene to heat-shocked *hsp70-Gal4/UAS-JHAMT RNAi* males completely rescued the courtship reduction (Fig 3C).

**Fig 4:**
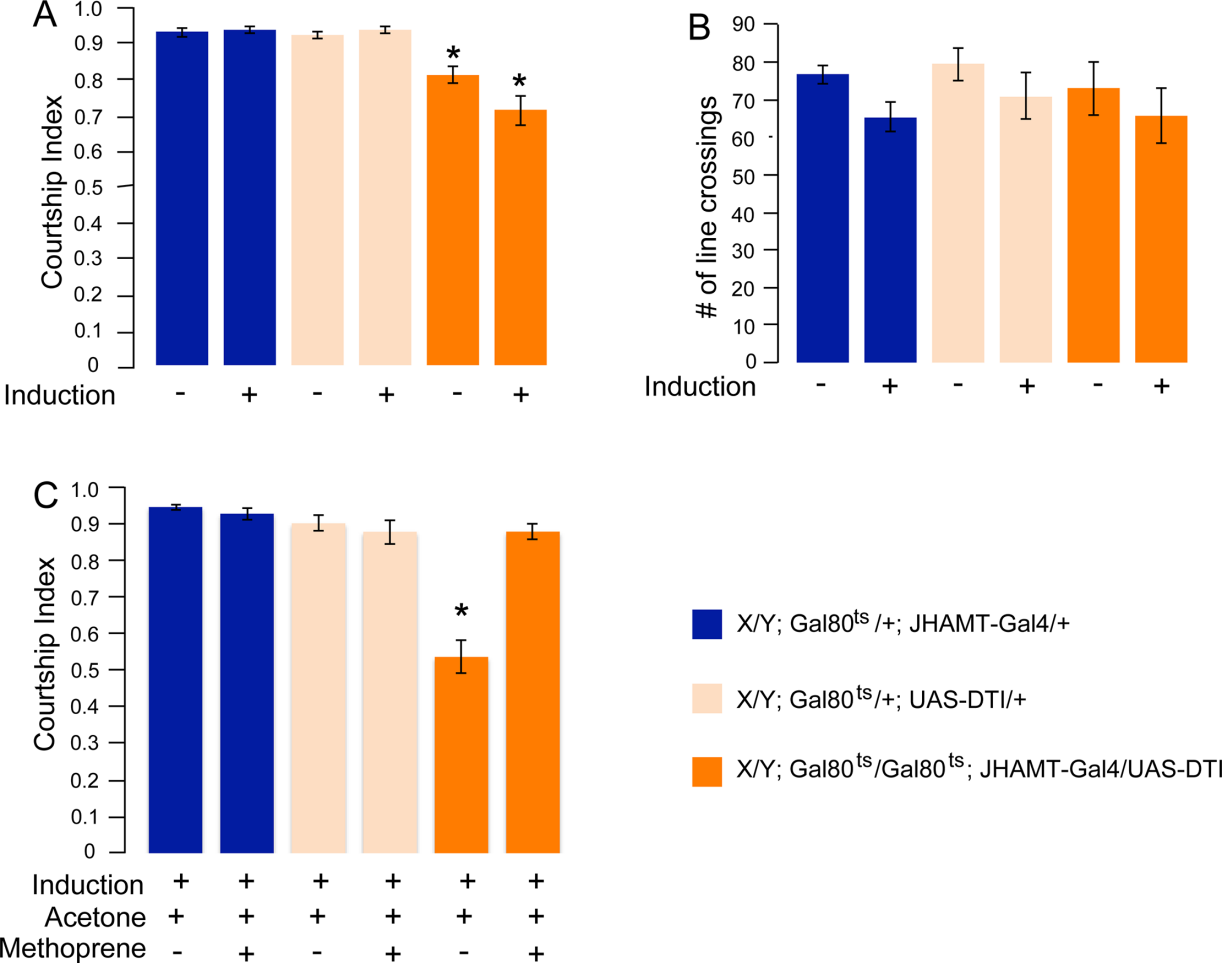
Conditional adult ablation of the corpora allata (CA) causes courtship defects that can be rescued by application of the JH analog Methoprene. The conditional Gal80^ts^ system was used to express UAS-DTI in adult males. The flies were created and maintained at 18°C. For induction, mature males were placed at 30°C for 2 days and then kept at room temperature for one day. The induced and un-induced experimental and control genotypes were subjected to courtship assay. Graphs show the courtship index CI (fraction of time males spend courting during the observation period) ± SEM of the indicated genotypes (A, C), or the performance of males in a control activity assay (# of line crossings ± SEM (B). Data were analyzed by ANOVA followed by Bonferroni multiple comparisons. (A) Courtship assay of induced and un-induced experimental *X/Y; Gal80*^*ts*^*/Gal80*^*ts*^*; JHAMT-GAL4/ UAS-DTI* and control genotypes *X/Y; Gal80*^*ts*^*/+; UAS-DTI/ +;* and *X/Y; Gal80*^*ts*^*/+; JHAMT-GAL4/+*. (N = 15). Experimental flies had significantly lower courtship than the controls (p<0.01). (B) Activity assay of the flies described in (A) (N = 10). (C) Methoprene in acetone or acetone alone was applied to induced males four hours prior to testing for courtship. Methoprene application completely rescued the courtship defect of experimental flies (P<0.001). (N = 20). (Un-induced (-), Induced (+).

These results strongly support an adult requirement for JH in the regulation of male courtship. *hsp70-Gal4* is expressed throughout the flies. Since JH is produced in the CA it is likely that the observed effect was due to JH reduction in this organ. To confirm this, we conditionally ablated the CA by expression of DTI, a modified version of Diphtheria toxin [20] and tested courtship and Methoprene rescue. Indeed, the same result was also observed when we conditionally ablated the CA, the place of JH synthesis, by the conditional adult expression of Diphtheria Toxin (DTI) (Fig 4). DTI expression in cells results in toxicity by the attenuation of protein synthesis [37]. Due to the high toxicity of DTI, conditional adult expression in the CA was performed in the presence of two copies of temperature-sensitive Gal80^ts^, using the Gal80^ts^/Gal4/UAS (TARGET) system [38]. At 18°C, Gal80^ts^ is functional and blocks the activity of Gal4. When flies are placed at 30°C, Gal80^ts^ becomes inactive and Gal4 is active. *Gal80*^*ts*^*/Gal80*^*ts*^*; JHAMT-Gal4/UAS-DTI* flies were grown at 18°C. Upon eclosion, males were kept at 18°C for maturation. They were then placed at 30°C for two days, followed by room temperature for 24 hours to allow expression of DTI in the CA. As shown in Fig 4A, induced expression of DTI in the CA results in a decrease in courtship similar to the one observed in *hsp70-Gal4/UAS-JHAMT-RNAi* knock down flies. A smaller but significant decrease was observed in the absence of induction. This is most likely due to a “leakiness” of Gal80^ts^ that allows small amounts of Gal4 to be made. As shown in Fig 4B, general activity was not affected in the experimental genotypes under un-induced or induced conditions. We next examined whether the courtship of CA- ablated flies could be rescued by application of Methoprene. Flies were treated as in (A), but in addition, Methoprene in acetone or acetone alone were applied 3 hours prior to courtship assay (Fig 4C). As before, the reduced courtship could be rescued by Methoprene application. These experiments confirm that efficient courtship is dependent on JH that is made in the CA.

## Discussion

The results described here demonstrate that expression of an RNAi targeting JHAMT, a key enzyme in the biosynthesis of JH, results in a reduction of male courtship. Reduction of JHAMT is predicted to lead to lowered JH levels in these animals. Consistent with this, we find that application of Methoprene, a JH analogue, is capable of rescuing the phenotype. Furthermore, a reduction in courtship is also observed when the corpora allata, the site of JH synthesis, are conditionally ablated in mature males by the expression of DTI. These findings demonstrate that JH is required for normal male courtship behavior and identify a direct role for JH in adult fly behavior. Importantly, all of our manipulations can be rescued by application of the JH analogue Methoprene shortly before performing courtship assays. Therefore, the observed courtship defects are not a consequence of permanent developmental defects. The importance of JH during development has long been recognized and is well studied. In adult flies, its important role in female egg maturation is well known, but we are just beginning to understand some of its adult behavioral functions. Bilen et al have recently shown that in females JH regulates the production of cuticular pheromones and the onset of mating [20]. Our experiments now demonstrate that physiologically correct adult levels of JH are required for normal male courtship. Even in flies that have been expressing a weak JHAMT-RNAi throughout development the rescue of the courtship defect can be achieved by application of Methoprene to mature males shortly before they were tested in a courtship assay, underscoring the importance of normal JH levels for courtship. It is unknown how JH regulates courtship. JH as a hydrophobic molecule is thought to require a hydrophilic carrier protein for its transport to target cells. JH has been shown to be bound by JHBPs in the hemolymph of many insects [39–41]. However, hemolymph JHBPs have not been identified in *Drosophila* yet. Intriguingly, Takeout and the proteins of the Takeout family have similarity to JHBPs [12, 42], and *takeout* mutants have courtship defect, but it is unknown whether Takeout proteins bind JH.

In this study we have shown that JH regulates male courtship behavior in adult courting males, demonstrating a novel adult function for this important insect hormone. These results add to our increasing understanding that although sex determination in flies is mostly cell-autonomous and sexual neuronal circuits are determined during development, soluble circulating factors from the fat body and Juvenile Hormone are important regulators of the behavior. Future studies will have to reveal how JH and these factors interact with the relevant neuronal circuits to influence courtship.
